# Tropism of *Plasmodium malariae* for young red blood cells and the bone marrow

**DOI:** 10.1080/22221751.2026.2731507

**Published:** 2026-09-07

**Authors:** Barbara Mouyama, Vivienne Fey, Dorenda J.A. Schoorl, Malika Serra-Hassoun, Fidelis Ambinintsoa, Barclaye Ngossanga, Leticia Hartmann, Ayola A. Adegnika, Artur Scherf, Mario Recker, Rolf Fendel, Steffen Borrmann, Sylvie Garcia

**Affiliations:** a Institut für Tropenmedizin, University of Tübingen, Tübingen, Germany; b Parasitology and Insect Vectors Department, Institut Pasteur, Université Paris-Cité, Paris, France; c Centre de Recherches Médicales de Lambaréné, Lambaréné, Gabon; d École Doctorale Complexité du vivant, Sorbonne Université, Paris, France; e Faculty of Biosciences, University of Heidelberg, Heidelberg, Germany; f Malaria and Neglected Tropical Diseases, German Center for Infection Research (DZIF), Partner site Tübingen, Tübingen, Germany; g Biology of Host–Parasite Interactions Unit, Institut Pasteur,Paris, France; h Centre for Ecology and Conservation, University of Exeter, Penryn, UK

**Keywords:** *Plasmodium malariae*, neglected, reticulocytes, bone marrow, humanized mice

## Abstract

Fundamental aspects of the biology of the third most prevalent human malaria species ***Plasmodium malariae***, including the basis for its capacity to establish chronic, low-density infections associated with debilitating quartan fevers, remain poorly characterized, hampering control efforts. Here, we combined ex vivo flowcytometric profiling of *P. malariae* isolates with infection studies in a recently developed humanized mouse model. We demonstrate a 5-fold higher tropism of *P. malariae* compared to *P. falciparum* for immature red blood cells, challenging a nearly century-old paradigm, and – for the first time – provide evidence for the parasite’s capacity to home to and infect host bone marrow. Collectively, these findings reveal key life cycle features of this enigmatic and neglected human malaria parasite.

## Introduction

*Plasmodium malariae*, one of the six protozoan species that cause malaria in humans is globally distributed across sub-Saharan Africa, parts of Southeast Asia, the Western Pacific, and South America [1]. The global burden of disease attributable to *P. malariae*, however, remains unknown and unlike *P. falciparum* and, to some extent, *P. vivax*, the World Health Organization does not publish species-specific estimates for *P. malariae* [2]. *P. malariae* infections are often underreported due to mild clinical presentations and the difficulty to correctly identify low-blood stage density infections by microscopy [8], particularly in regions with co-endemic, typically much higher-density *P. falciparum* infections. Isolated, small-scale molecular epidemiological studies have documented both a marked increase in *P. malariae* prevalence across endemic regions [3–5] and prevalence rates of >20% by qPCR [6]. There is even less systematic data on life-threatening manifestations and mortality, but the consensus is that these are rare events [7]. The parasite follows a quartan fever pattern, with febrile episodes occurring every 72 hours, reflecting its prolonged intra-erythrocytic replication cycle as compared to other human malaria parasites with 48-hour cycles [14]. Unlike the other two non-*falciparum* human *P. vivax* and *P. ovale*, *P. malariae* does not form hypnozoites in the liver, but its long-term persistence, sometimes over decades, without symptoms, can give rise to delayed malaria episodes [8–13].

*P. malariae* remains understudied compared with other human malaria parasites, in part because it cannot be continuously cultivated in vitro and there is also no experimental animal model system. A key unresolved question concerns the propensity of *P. malariae* for low-density infections [14], which could be related to a comparatively narrow specificity for invading only a sub-population of red blood cells (RBCs). While preferential tropism for mature, even old, erythrocytes has been widely accepted for decades [14–16], multiple confounding factors may underlie this apparent preference for older RBCs, indicating the need to re-evaluate this paradigm [17]. First, the delay between sample collection and analysis may have allowed reticulocytes to mature into normocytes, potentially biasing observations towards a preferential infection of older RBCs [17]. Second, the low proportion of circulating reticulocytes could have impeded the direct microscopic visualization of infected reticulocytes.

Here, we revisited this dogma and addressed these limitations by using adapted flowcytometric methods. We report that *P. malariae* predominantly infects reticulocytes (CD71^+^ RBCs) in human infections. Moreover, in humanized mice that support efficient human haematopoiesis and RBC development [18], we demonstrate that *P. malariae* can not only home to the bone marrow (BM) but also infect CD71^+^ erythroid cells within this organ. This finding further indicates that extracellular reservoirs, previously identified as niches for both *P. falciparum* and *P. vivax* [19,20], also exist for *P. malariae*.

## Material and methods

### *P. malariae* isolates

The identification of *P. malariae* infections was done as part of a cross-sectional, household-based surveillance of *P. malariae* in both human and mosquito populations in the Moyen-Ogooué province, Gabon [21]. This study also included community-based screening to obtain *P. malariae* isolates with gametocytes for studying transmission to mosquito colonies. Here, we used this screening effort to identify asexual *P. malariae* infections. All procedures on fresh isolates (thin and thick Giemsa blood smears, centrifugation-based leukocyte depletion, flow cytometry, cryopreservation for both cells and RNA extraction) were performed at the Centre de Recherches Médicales de Lambaréné (CERMEL). All samples were tested as mono-infected by one *Plasmodium* *spp* using species-specific qRT-PCR assays recently optimized by our team [21,22]. The study protocol was approved by the Gabonese National Ethics Committee (040/2018/SG/CNE), the institutional review board of CERMEL (CEI-014/2018), and the ethics committee of the Institute of Tropical Medicine, University of Tübingen, Germany (010/2018BO2). Three clinical *P. vivax* samples collected in North-western Brazil (Amazonian region of Acre) were leukocyte-depleted and cryopreserved in liquid nitrogen [23]. Study protocols have been approved by the Institutional Review Board of the Institute of Biomedical Sciences, University of São Paulo, and by the National Human Research Ethics Committee of the Ministry of Health of Brazil (CAAE: 64767416.6.0000.5467). All patients provided written informed consent.

### RNA extraction and *Plasmodium species* discrimination by qRT-PCR

All procedures were performed according to a recently optimized protocol established by our team in CERMEL [21,22]. Two to five mL of total blood from patients were collected into PAXgene-stabilized blood (BD, #12937706). Total RNA (0.28–65.20 ng/µL) was then extracted using the Mag-Bind® Total RNA 96 Kit (Omega Bio-Tek, #M6731-01) on a KingFisher® Flex instrument (Thermo Scientific), following the manufacturer’s instructions. A one-step quantitative reverse transcription PCR (qRT-PCR) assay was used to differentiate *P. falciparum* and *P. malariae* mono-infections in separate reactions using 18S rRNA-species-specific primers and probes, using 1 µL RNA, 5 µL SensiFAST™ One-Step Mix (Bioline, #BIO-76005), 0.1 µL transcriptase, and species-specific primers and probes (0.4 µL of each 10 µM primer; 0.2 µL of 10 µM probe). The primer and probe sequences were: Plas-Forward: 5’-GCT TAG TTA CGA TTA ATA GGA GTA GCT TG-3’, Plas-Reverse: 5’-GAA AAT CTA AGA ATT TCA CCT CTG ACA-3’, and Pf-Probe: 5’-[FAM] TCT GAA TAC GAA TGT C [MGBEQ] -3’; PmT-Forward : 5’-GGT GTT GGA TGA TAG AGT AA-3’, PmT-Reverse: 5’-CCC AAA GAC TTT GAT TTC TC-3’, and PmT-Probe: 5’-[HEX] AGG AAG CTA TCT AAA AGA AAC ACT CAT [MGBEQ]-3’. Amplification was performed on a LightCycler® 480 II (Roche) as follows: 45°C for 20 min; 95°C for 5 min; 50 cycles of 95°C for 15 s, and 60°C for 60 s. Samples were run in triplicate, and a quantification cycle (Cq) value below 35 was considered positive.

### Flow cytometric analysis of infected red blood cells from clinical isolates

One µL of leukocyte-depleted fresh red blood cells from mono-infected *P. malariae* (n = 4), uninfected blood samples (n = 3), *P. falciparum* mono-infected isolates (n = 10), and *P. vivax* cryopreserved mono-infected clinical isolates from Brazil (n = 3) were washed and stained with Hoechst 33342 (16.2 µM; Thermo Fisher, #H3570) for 30 min at room temperature. Cells were then stained with fluorescently conjugated antibodies against CD45 (FITC; BioLegend, #368508) and CD71 (PE-Vio615; Miltenyi Biotec, #130-115-036). Samples were acquired on a BD Symphony A1 cytometer at the field site. A minimum of 1 million events were acquired for stained samples. Data were analysed using FlowJo software (v10.6.1, BD). The gating strategy is shown in Supplementary Figure S1.

### Imaging flow cytometry

One µL RBC pellets from cryopreserved isolates were stained with Hoechst 33342, anti-CD45-FITC, anti-CD71-PE-Vio615, and anti-CD235a-AlexaFluor647 (#349130, BioLegend). HIS-HEry BM cells were fixed in 4% PFA/0.075% glutaraldehyde, stained with DAPI, anti-CD235a-FITC (#349104, Biolegend), and anti-CD71-AlexaFluor700 (#334130, Biolegend). Data were analysed using IDEAS software (v6.2). To visualize infected cells, cryopreserved *P. malariae* isolates, and HIS-HEry BM cells were analysed on an ImageStream ISX MkII (Amnis Corp.). Supplementary Figures S2–S4 show the gating strategy.

### Generation of humanized mice (HIS-HEry)

Human Immune System Human Erythrocyte (HIS-HEry) mice were generated as described [18]. Briefly, CD34+ haematopoietic stem and progenitor cells (HSPCs) were isolated from human cord blood (AP-HP, Hôpital Saint-Louis, authorization AC-2016-2759) using the EasySep™ Human CD34 Positive Selection Kit II (Stemcell Technologies, #17856). 1.7–1.9 × 10^5^ CD34^+^ cells were injected intrahepatically into 3–4-day-old immunodeficient CH1-2hSaW41HHH neonates. Human CD45^+^ chimerism was assessed at 3 months post-engraftment by flow cytometry. Levels of human BM CD235a^+^ erythroid cells in HIS-HEry mice ranged from 7% to 40%, among which 35–74% were CD71^+^ cells. Mice were euthanized by progressive inhalation of CO_2_. All animal experiments were approved by the Pasteur Institute Animal Ethics Committee (CETEA dap230081).

### Infection of HIS-HEry mice with *P. malariae*

Mice received an intravenous injection of 200 µL clodronate liposomes (Liposoma BV) one day before infection and every 5 days thereafter. Mice were infected via retro-orbital injection with 2.5 × 10⁶ to 5 × 10⁶ *P. malariae*-infected RBCs from thawed field isolates. At day 9 post-infection, mice were euthanized, and peripheral blood and BM were harvested for analysis.

### Parasite quantification in HIS-HEry mice

Total RNA from blood and BM of HIS-HEry mice was extracted using NucleoZol® (Machery-Nagel, #740404.200) and the NucleoSpin® RNA kit (Machery-Nagel, #740406.50). RNA was quantified by NanoDrop and reverse-transcribed into cDNA with SuperScript™ II Reverse Transcriptase (Invitrogen, #18064-022). A plasmid standard (pLN-Pm18S) containing a 121-bp fragment of the *P. malariae* 18S rRNA gene was generated by In-Fusion® HD cloning (Takara Bio) into a circular pLN-backbone vector. Serial dilutions of the plasmid (5–5.10^9^ copy/µL) were used as standards in qPCR assays. Twenty ng RNA per sample and plasmid standards were submitted to qPCR using SsoAdvanced™ Universal SYBR® Green Supermix (Bio-Rad, #1725271) to detect the *P. malariae* 18S rRNA gene using the primers PmT-Forward/Reverse. Amplification conditions on a CFX96 Touch system (Bio-Rad) were: 95°C for 6 min; 50 cycles of 95°C for 15 s, 60°C for 20 s, 72°C for 30 s; followed by a melt curve analysis. Samples with Cq values < 38 (*Pm18S*) were considered positive. Copy numbers were calculated from the standards and reported as the numbers of CD235a^+^ RBCs calculated from flow cytometric analyses.

### Statistical analysis and mathematical modelling

In Figure 1(a), statistical comparisons between the three groups were performed using a mixed-effects logistic regression. In Figure 3(b), statistical comparisons between the two groups were performed using a Wilcoxon signed-rank test. All *P*-values < 0.05 were considered statistically significant. All analyses were conducted using R (version 4.2.2) and RStudio software (version 2022.02.3)

**Figure 1.**
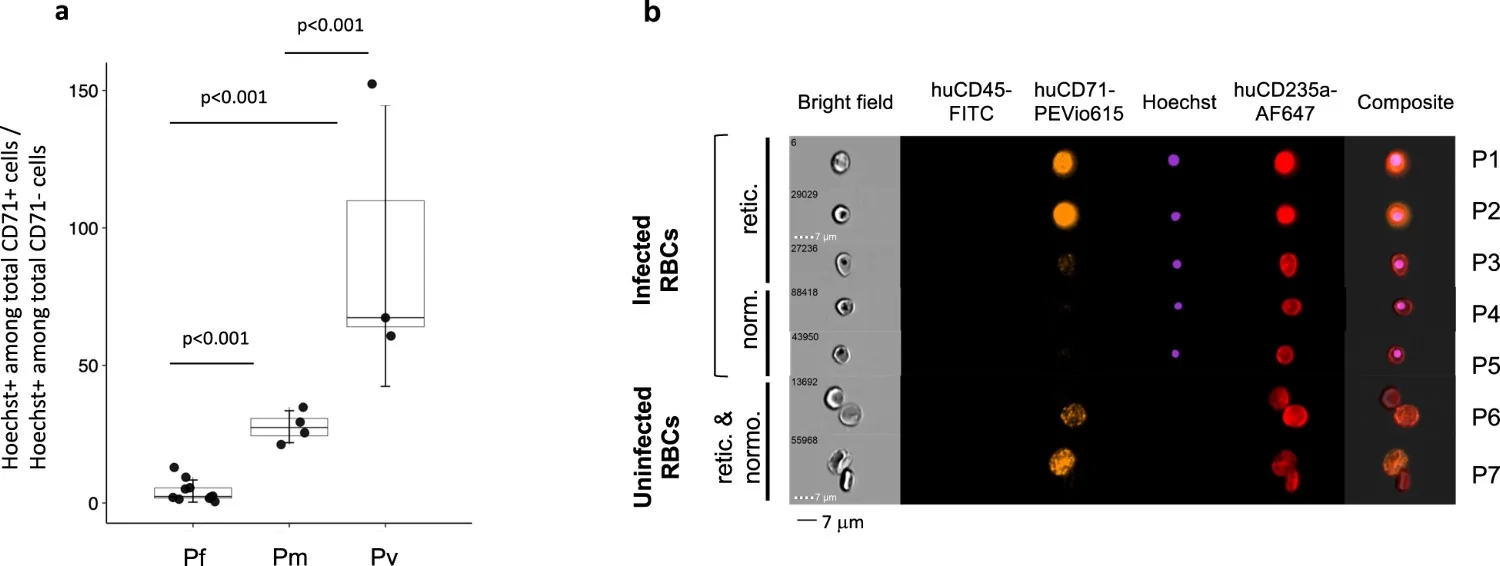
**| Peripheral blood isolates from infected patients show *P. malariae-*infected reticulocytes.** Peripheral blood isolates from *P. malariae* (n = 4), *P. falciparum* (n = 10), and *P. vivax* (n = 3) mono-infected patients were analysed by Flow cytometry (a) and Flow Imaging (b). **a –** Box plot shows the ratio of immature CD45^-^ CD71^+^ Hoechst^+^ versus mature CD45^-^ CD71^-^ Hoechst^+^ infected cells. **b –** Representative ImageStream profiles of *P. malariae*-infected isolates, stained with leukocytic marker CD45 in green, reticulocyte CD71 marker in orange, parasite nuclei coloured with Hoechst in violet, CD235a RBC marker in red and composite image (right merge). Seven different images are shown: P1–P2 correspond to CD45^-^ CD235a^+^  CD71^hi^ Hoechst^+^ infected young reticulocytes; P3 corresponds to CD45^-^ CD235a^+^  CD71^lo^ Hoechst^+^ infected old reticulocyte; P4–P5 correspond to CD45^-^ CD235a^+^  CD71^-^ Hoechst^+^ infected mature RBCs; P6–P7 corresponds to mixed CD45^-^ CD235a^+^  CD71^-^ and CD71^+^ Hoechst^-^ uninfected reticulocytes and mature RBCs; Scale bar: 7 µm.

To estimate relative reticulocyte tropism while accounting for differences in erythrocytic cycle length, we used a simple kinetic model of reticulocyte maturation. We assumed that CD71 expression is lost exponentially following the release of reticulocytes into the circulation, with a loss rate *μ* corresponding to CD71 half-life. Under this model, the probability that an infected reticulocyte remains CD71^+^ during observation decreases as $e^{-\mathrm{\mu T}}$, where *T* is the duration of the erythrocytic cycle (*P. vivax: T* *=* 2 days, *P. malariae*: *T* = 3 days).

Let *r_v_* and *r_m_* denote the observed ratios of CD71^+^ to CD71^-^ infected erythrocytes for *P. vivax* and *P. malariae*, respectively. Assuming erythrocytic cycle lengths of 2 and 3 days, the relative reticulocyte preference *k_v_* / *k_m_* was estimated as

$$\frac{k_{v}}{k_{m}}=\frac{r_{v}}{r_{m}}e^{-\mu }$$

where the exponential term corrects for the additional day available for CD71 loss during the longer *P. malariae* erythrocytic cycle.

A sensitivity analysis was performed across a range of plausible CD71 half-lives to assess the effect of uncertainty in reticulocyte maturation kinetics on the inferred relative tropism.

## Results

### *P. malariae* preferentially infects reticulocytes in human blood

To examine the potential tropism of *P. malariae* for a specific subset of RBCs, we analysed the frequencies of mature versus young *P. malariae*-infected RBCs in the peripheral blood samples of Gabonese volunteers. For comparison, we also evaluated *P. falciparum* mono-infected isolates from the same Gabonese population, and 3 *P. vivax* isolates originating from Brazil. Study participants ranged in age from 6 to 52 years; 9 of 14 were female and 4 presented with fever (38–39°C) (Supplementary Table). Parasitemia ranged from 232 to 29,000 parasites/μl for *P. falciparum*, from 754 to 4,756 parasites/μl for *P. malariae,* and from 750 to 2250 parasites/μl *for P. vivax* (Supplementary Table). We then used a flow cytometry approach that allowed us to evaluate the tropism of *P. malariae* for CD71^+^ reticulocytes compared to CD71^-^ mature normocytes. *Plasmodium* infection was assessed via Hoechst staining of parasite DNA in enucleated immature CD71^+^ (reticulocytes) and mature CD71-^-^ CD45^-^ RBCs after exclusion of CD45^+^ nucleated Hoechst^+^ leukocytes (see Supplementary Figure S1a for the gating strategy). We detected the presence of Hoechst^+^ parasitized RBCs in all infected samples, while no signal was observed in blood from *Plasmodium*-negative individuals, confirming the validity of our staining (Supplementary Figure S1b). Our analysis showed the presence of parasites in both CD71^+^ immature and CD71^-^ mature RBCs for all parasites (Supplementary Figure S1b). However, and in accordance with the established restriction of *P. vivax* for reticulocytes [24], the ratio of parasite-infected CD71^+^ reticulocytes versus CD71^-^ mature RBCs was about 18-fold higher for *P. vivax* than for *P. falciparum* (93.5 ± 51.1 versus 4.34 ± 4.03 expressed as mean ± SD) (Figure 1(a)). Remarkably, we also found a 5-fold higher ratio for *P. malariae* compared to *P. falciparum* (27.7 ± 5.81 versus 14.34 ± 4.03 expressed as mean ± SD), i.e. only 3-fold lower than for *P. vivax* (27.7 ± 5.81 versus 93.5 ± 51.1 expressed as mean ± SD), demonstrating the propensity of *P. malariae* to infect young RBCs. To validate the presence of *P. malariae* in young CD71^+^ RBCs, we analysed the same samples by flow imaging (see Supplementary Figure S2 for the gating strategy). This approach confirmed our ex vivo observations showing CD235a^+^ CD45^-^ Hoechst^+^ infected RBCs with both high (young reticulocyte; P1–P2), or low (old reticulocyte; P3) CD71 fluorescence intensities, and with no CD71 staining (mature RBCs; P4–P5) (Figure 1(b)). Collectively, our results demonstrate a pronounced preference of *P. malariae* blood stages for young reticulocytes.

Our experimental data, showing that *P. vivax* exhibited a higher observed frequency of infection in CD71^+^ erythrocytes than *P. malariae*, however, is confounded by the one-day difference in erythrocytic cycle length. Because infected reticulocytes continue to mature during parasite development, a larger proportion of *P. malariae*-infected reticulocytes are expected to lose CD71 expression before observation. After correcting for this effect using a simple reticulocyte maturation model (see Methods), the inferred difference in intrinsic reticulocyte preference between the two species was substantially smaller than suggested by the observed CD71^+^/CD71^-^ ratios alone (Figure 2).

**Figure 2.**
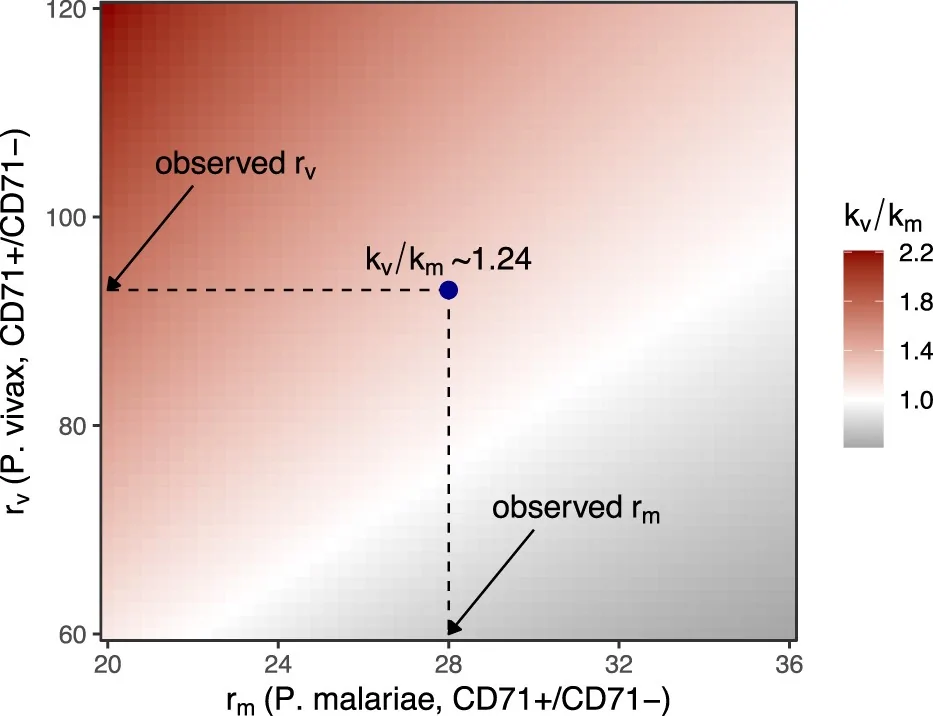
**| Inferred relative reticulocyte preference as a function of observed CD71^+^/CD71^-^ ratios of *P. vivax* (*r_v_*) and *P. malariae* (*r_m_*)**. Colours indicate the inferred ratio of intrinsic reticulocyte preference (*kv/km*) after correcting for the one-day difference in intra-erythrocytic cycle length between the two species. Values greater than 1 indicate stronger reticulocyte preference by *P. vivax*, whereas values less than 1 indicate stronger reticulocyte preference by *P. malariae*. Results shown based on an assumed mean CD71 lifetime of 1 d after release or reticulocytes from the BM, corresponding to a half-life of ∼17 h.

**Figure 3.**
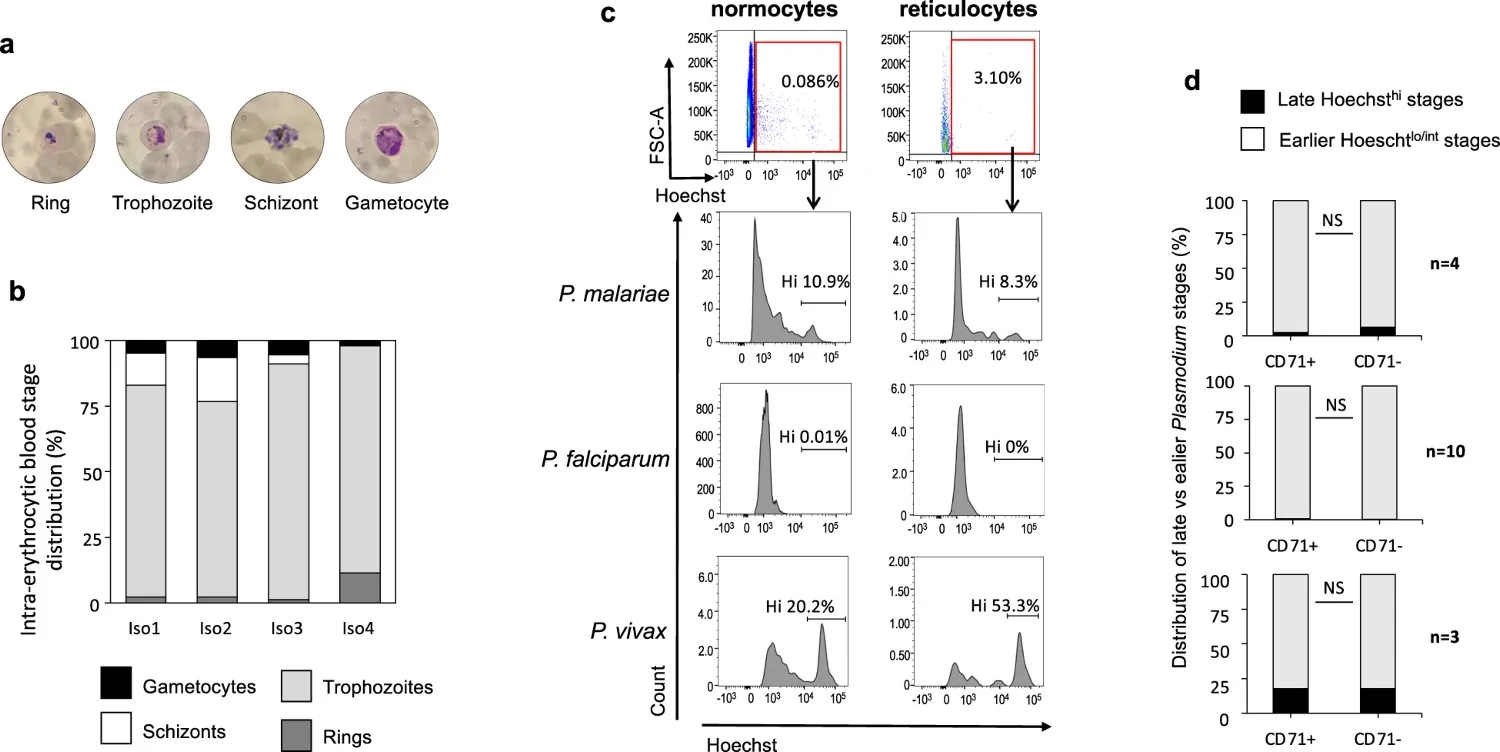
**| *P. malariae-*infected reticulocytes from peripheral blood isolates can host late-stage parasites.** Parasite stages were studied on the same peripheral blood isolates than in Figure 1. **a –** Giemsa smear microscopic representative images showing the concomitant presence of ring, trophozoite, schizont, and gametocyte blood stages in *P. malariae*-infected patients; **b –** Bars show the distribution of all stages detected by microscopy in each *P. malariae* isolate, **c –** Hoechst levels were used to quantify late and early stage parasites by flow cytometry. Hoechst vs size (FSC-A) upper dot plots show the gating of Hoechst^+^ normocytes (left) and Hoechst^+^ reticulocytes (right). Percentages of Hoechst^hi^ cells among Hoechst^+^ normocytes (left histograms) and reticulocytes (right histograms) are shown for *P. malariae*, *P. falciparum*, and *P. vivax*-infected isolates. **d –** Bars show proportions of late Hoechst^hi^ (black) vs earlier Hoechst^lo/int^ (grey) parasites in CD71^+^ (left bars) vs CD71^-^ (right bars) RBCs for pooled *P. malariae* (n = 4)*, P. falciparum* (n = 10), *and P. vivax* (n = 3) infected isolates. NS: non significant.

The estimated relative tropism, based on the observed *Pv:Pm* invasion preference ratio, varied with the assumed CD71 half-life. Importantly, however, across a biologically plausible range (0.5–1.6 days) it remained consistently and significantly lower than the uncorrected ratio (3.4), up to a point where the inferred relative tropism was consistent with Supplementary Figure S3 .

### Presence of both young and late *P. malariae* blood stages in reticulocytes suggests early infections of reticulocytes in human *P. malariae* infections

To get insights into the dynamics of *P. malariae*, we then investigated the degree of parasite blood stage differentiation in both mature and immature RBCs. As the *P. malariae* blood stage cell cycle takes 3 days, we expected to observe very few fully differentiated, multinuclear schizonts in reticulocytes, which typically complete their maturation process within 1-2 days after their release from the BM (associated with a loss of CD71 staining) [25]. Microscopic analyses showed the presence of all asexual blood stages as well as gametocytes in *P. malariae*-infected donor blood (Figure 3(a)), with a predominance of trophozoites (Figure 3(b)). Because the intensity of parasite DNA staining correlates with intra-erythrocytic parasite replication [26], we then asked whether both early and late stages could be found in mature and immature RBCs using Hoechst DNA staining intensities in both CD71^+^  and CD71^-^ RBCs (Figures 3(c) and (d)). Most parasite-infected RBCs from *P. falciparum* isolates expressed low Hoechst levels consistent with ring and young trophozoite stages in the absence of late-stage forms due to tissue sequestration. In contrast, *P. vivax*-infected cells contained both early and later stage parasites, consistent with the asynchronicity observed for this parasite *in vivo*. Interestingly, we observed that high Hoechst expression levels could not only be found in CD71, but also in CD71^+^ RBCs in *P. malariae* (Figures 3(c) and (d)). This strongly suggests that reticulocytes had been infected earlier during their development, potentially already within the BM, the main site of erythropoiesis in humans.

### *P. malariae* can home to and infect BM CD71 +  cells in a humanized HIS-HEry mouse model

Due to the difficulty of accessing human BM biopsies, we leveraged a new humanized HIS-HEry mouse model able to produce human RBCs upon human haematopoietic progenitor engraftment. We previously showed that HIS-HEry mice could support *P. vivax* infection and sexual differentiation in the BM upon inoculation of infected blood [18]. We injected 2 groups of HIS-HEry mice (total of *n *= 9) reconstituted with human haematopoietic progenitors from 2 different cord blood samples, with 2 different *P. malariae* isolates (Figure 4(a)). Nine days later, the blood and BM samples from infected mice were analysed to quantify *P. malariae* infections using our species-specific 18S rRNA qRT-PCR. Parasites were detected in the blood of 8 of 9 mice and in the BM of all 9 mice. Although not significant, parasite burden tends to be higher in the BM compared to circulating blood (Figure 4(b)). To characterize the maturation status of parasitised RBCs, we analysed by Flow-Imaging BM RBCs from infected mice (Figure 4(c)). The gating strategy is detailed in Supplementary Figure S4. We observed the presence of DAPI^+^  infected cells among CD71^+^ CD235a^+^  immature BM RBCs (Figure 4(c) P1–P4). We also provide images of nucleated DAPI^+^  CD71^+^ CD235a^+^  erythroblasts (Figure 4(c), P8–P9), which possess a significantly larger nucleus that can be easily distinguished from parasite nuclei (Figure 4(c) P1–P4). This was further confirmed by qRT-PCR on sorted BM CD235a^+^  CD71^+^  and CD235a^+^  CD71^-^, which both contain *P. malariae*-specific 18S rRNA (Supplementary Figure S5). These results extend our observations from natural *P. malariae* infections by confirming *P. malariae* infection of immature RBCs and revealing that the parasite can home to and selectively infect young RBCs in the BM.

**Figure 4.**
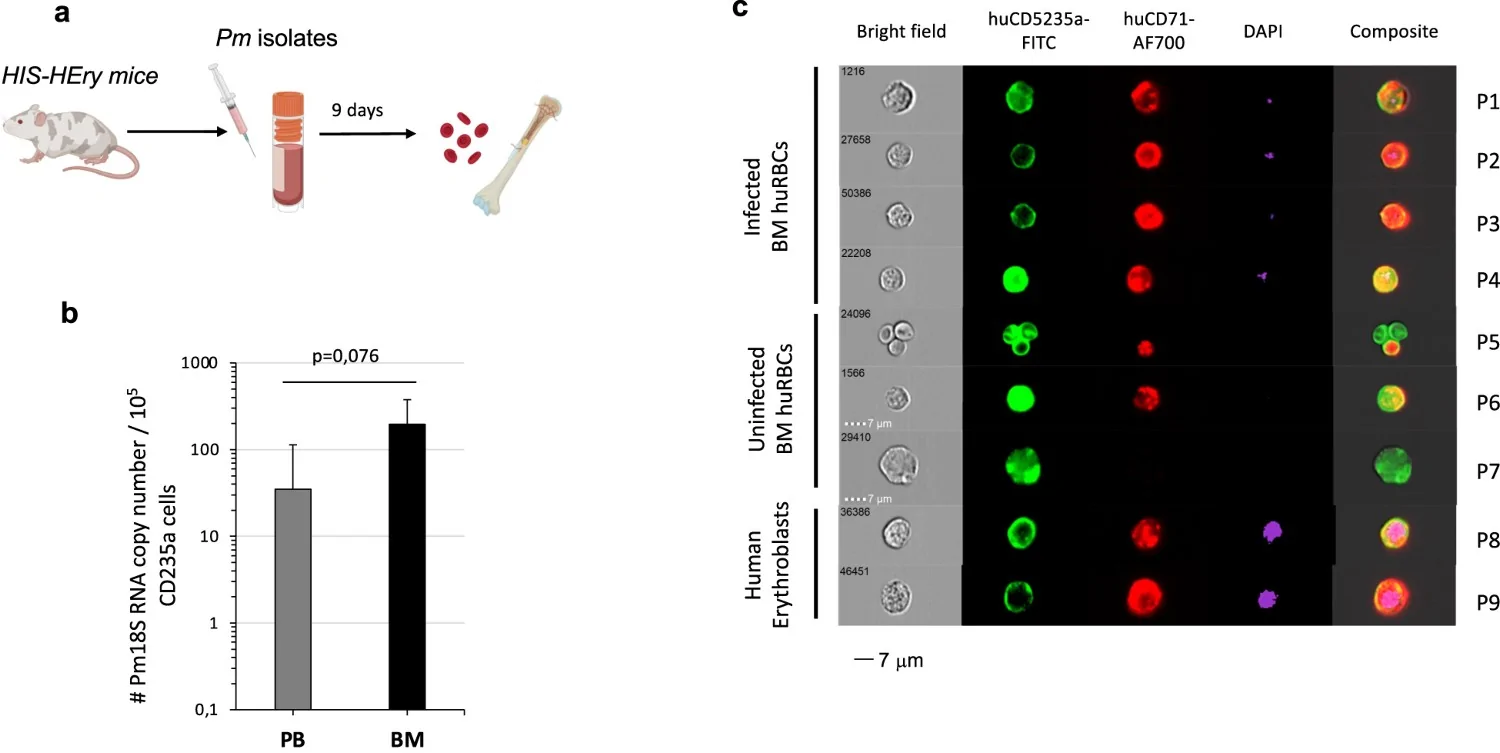
***P. malariae* can home and infect BM in HIS-HEry chimeras. a –** HIS-HEry chimeras (n = 9) were infected with *P. malariae*-infected isolates, and blood and BM were sampled 9 days post-inoculation. **b** – Bars show the numbers of Pm18S rRNA copies per 10^5^ human CD235a^+^  blood and BM cells for the 9 infected mice. Mean Pm18S rRNA copy numbers are for blood =  37.01±79.02 and BM =  196.06±181.52. **c –** ImageStream pictures of BM RBCs from *P. malariae*-infected-HIS-HEry humanized mice. Cells were stained with human CD235a RBC lineage marker in green, human reticulocyte CD71 marker in red, parasite nuclei coloured with DAPI in violet, and composite image (right merge). Nine different images from different infected mice were shown: P1–P4 correspond to infected CD235a^+^  CD71^+^ DAPI^+^  infected reticulocytes; P5 corresponds to a mix of an uninfected CD235a^+^  CD71^+^ DAPI^-^ reticulocyte and uninfected CD235a^+^ CD71^-^ DAPI^-^ normocytes; P6 corresponds to a uninfected CD235a^+^ CD71^+^ DAPI^-^ reticulocyte; P7 corresponds to an uninfected CD235a^+^  CD71^-^ DAPI^-^ normocyte; P8 and P9 correspond to DAPI^+^  CD235a^+^  CD71^+^  nucleated erythroblasts. Scale bar: 7 µm.

## Discussion

Malaria control is currently mainly targeting *P. falciparum* and *P. vivax. Plasmodium malariae,* the third most important human malarial parasite, even though widely distributed and in some regions highly prevalent, receives far less attention. This is mainly due to the perception of *P. malariae* as a cause of benign, i.e. not life-threatening, fever episodes with spectacular quartan chronicity in non-immune individuals [14]. Chemotherapies are rarely evaluated in *P. malariae* patients, nor is there currently any vaccine candidate for *P. malariae,* and advances are currently hindered by significant gaps in our understanding of key life history traits of this neglected parasite. For instance, a long-standing dogma holds that *P. malariae* exhibits a tropism for circulating mature, even old, erythrocytes [14–16]. Our study overturns this prevailing paradigm by showing that *P. malariae* preferentially infects CD71^+^  reticulocytes, both in clinical isolates from Gabonese volunteers and in a humanized BM mouse model, highlighting the BM as an important tissue niche for parasite development.

Using a combination of flow cytometry and imaging flow cytometry, we unequivocally demonstrated reticulocyte infection across all four analysed isolates from *P. malariae* mono-infected patients, in contrast to a recent study from Mali [27] and despite the limitation of small sample size of the blood isolates used in this study. Using a simple mathematical model, we further demonstrated that *P. malariae* exhibits a comparable degree of tropism for reticulocytes to *P. vivax*. In both cases, the restriction for reticulocytes, however, appears to be incomplete in our assays – most likely due to (i) the naturally occurring, gradual loss of CD71 during reticulocyte maturation within 1–2 days in the periphery [25] and (ii) at least as demonstrated for *P. vivax*, an accelerated, post-invasion shedding of CD71 within 3–6 h [24]. Interestingly, the *P. malariae* genome encodes orthologues for both PvRBP2a and PvRBP2b [27], two *P. vivax* ligands that bind to the reticulocyte-specific receptors CD98 and CD71, respectively [28,29]. It is therefore tempting to speculate that, similar to *P. vivax*, *P. malariae* may rely on either or both receptors to preferentially target young RBCs.

Microscopic analysis of *P. malariae* isolates from mono-infected individuals revealed the presence of all parasite blood development stages, including late-stage schizonts and a predominance of trophozoites. Infections did not appear to be synchronized, in line with the seemingly complete absence of strict quartan chronicity of fever episodes in malaria endemic populations. This finding was confirmed by flow cytometry of RBCs that contained increasing intensities of the DNA marker Hoechst corresponding to parasites advancing through the cell cycle. More surprisingly, this was also observed for CD71^+^  immature RBCs. Considering that CD71^+^  reticulocytes mature into CD71^-^ normocytes within 1–2 days after exiting the BM and that the asexual cell cycle of *P. malariae* takes 3 days, it led us to hypothesize that initial infection may occur within the BM, the site of erythropoiesis. Alternatively, this finding could also be explained by a delay in the maturation of reticulocytes by *P. malariae*, in contrast to what had been described for *P. vivax* [24]*.*

Using humanized mice that support human erythropoiesis [18], we confirmed that *P. malariae*-infected cells from patients could maintain the infectious cycle within the BM for at least nine days. We further demonstrated that parasite load was not significantly different between the BM and the blood of mice, and that this increase was associated with infection of BM reticulocytes. Since the parasite has a 72 hour replication cycle, the detection of parasite-infected BM cells 9 days after inoculation strongly supports de novo infections occurring in the BM of HIS-HEry mice. While a BM reservoir is well-established for *P. vivax* [20] and *P. falciparum* [19], its role in *P. malariae* infection has not been previously explored. Our findings suggest that the BM serves as a niche for this species.

In contrast to what we previously reported in HIS-HEry mice for *P. vivax* [18], we did not observe any gametocytes in the BM of *P. malariae*-infected mice. However, this may reflect the relatively short post-inoculation observation time frame, as this parasite may undergo a prolonged sexual differentiation process [30,31]. Therefore, we cannot currently speculate on the BM as a site for sexual development, as for *P. vivax* and *P. falciparum*. Another more compelling hypothesis is that the BM may constitute a specialized niche for schizonts, which are largely absent from peripheral blood and may preferentially home to this organ. This hypothesis is supported by our data supporting de novo infection of BM reticulocytes, the scarcity of late-stage parasites in peripheral blood in Gabonese volunteers, and a recent case report of *P. malariae* schizonts in the BM of an Indonesian patient with undetectable peripheral parasitaemia [32].

If confirmed in future studies also involving different geographical locations, the apparent restriction of *P. malariae* for reticulocyte invasion may carry significance for long-standing epidemiological observations. For instance, it could explain the low maximum density of untreated *P. malariae* mono-infections below 10,000/µL [14], i.e. not exceeding the number of reticulocytes in circulation. It could also have therapeutic relevance. Despite limited clinical evaluation of antimalarials against *P. malariae*, field studies have documented significant treatment failures with first-line artemisinin-based combination therapies and chloroquine [33–35]. While the extent of drug resistance of *P. malariae* remains poorly characterized, these observations suggest an evolving threat to elimination efforts that parallels the resistance trajectories previously documented in *P. falciparum* and *P. vivax*, including antifolate resistance [36]. In this context, parasite persistence in the BM may represent an additional therapeutic challenge, potentially compromising drug penetration or activity in this niche [37].

In conclusion, our study redefines the host-cell tropism of *P. malariae*, identifying the BM as a potential sanctuary that sustains infections. We also establish an experimental HIS-HEry mouse model to study the biology of this pathogen. Together, these findings reshape our understanding of this enigmatic human malaria parasite species and may help develop improved diagnostics and therapies aimed at achieving complete eradication of malaria.

## Supplementary Material

Revised Supplementary Material.docx
