# Supplementary material for: Tropism of Plasmodium malariae for young red blood cells and the bone marrow
Source: Emerg Microbes Infect. 2026 Sep 7;15(1):2731507. doi: 10.1080/22221751.2026.2731507 (PMC13651831; doi:10.1080/22221751.2026.2731507)
Supplement: Supplementary file 1 [file TEMI_A_2731507_SM4986.docx]

| ***Plasmodium spp*. isolates** | **qRT-PCR** | **Parasitemia GIEMSA**  **(p/uL)** | **Volunteers parameters** | | | |
| --- | --- | --- | --- | --- | --- | --- |
|  |  |  | **Sexe** | **Age** | **T°C** | **Localization (Gabon, except Pv)** |
| Iso 1 | Pf | 232 | M | 10 | 37 | Siat |
| Iso 2 | Pf | 29000 | F | 15 | 37,5 | Bindo |
| Iso 3 | Pf | 9600 | F | 6 | 37,5 | Lambarene |
| Iso 4 | Pf | 1682 | F | 52 | 36,9 | Bindo |
| Iso 5 | Pf | 19372 | F | 15 | 37.2 | Bindo |
| Iso 6 | Pf | 6786 | F | 15 | 37 | Petit Paris 3 |
| Iso 7 | Pf | 34249 | F | 10 | 38.2 | Abongo |
| Iso 8 | Pf | 14645 | M | 24 | 39 | Abongo |
| Iso 9 | Pf | 19662 | M | 11 | 36.6 | Benguie 4 |
| Iso 10 | Pf | 10786 | M | 40 | 38 | Moussamoukoungou |
| Iso 11 | Pm | 754 | F | 17 | 36,7 | Moussamoukoungou |
| Iso 12 | Pm | 1392 | M | 27 | 37 | Benguier |
| Iso 13 | Pm | 677 | F | 7 | 36,9 | Paris Bifoun |
| Iso 14 | Pm | 4756 | F | 47 | 38 | Abongo |
| Iso 15 | Pv | 2250 | NC | NC | NC | Acre, Brazil |
| Iso 16 | Pv | 750 | NC | NC | NC | Acre, Brazil |
| Iso 17 | Pv | 1500 | NC | NC | NC | Acre, Brazil |

**Supplementary Table: Patients’ information.** The table lists the following variables for patients tested by qRT-PCR for *Plasmodium* spp : parasitemia, sex, age, body temperature, and location at the time of sampling. Data are provided for *P. malariae* (n=4), *P. falciparum* (n=10), and *P. vivax* (n=3). NC = non-communicated.

**Figure S1**

**CD71± RBCs gating**

SSC-A

FSC-A

FSC-A

Hoechst

**CD71+**

FSC-A

Hoechst

**Single cells**

FSC-H

FSC-A

**CD45- cell exclusion**

FSC-A

CD45

**leukocytes**

CD71

FSC-A

**CD71-**

**a**

**b**

**Fig.S1**: **Gating strategies of the flowcytometric characterization of parasitized RBCs in blood samples**. **a**- An example of gating strategy for the quantification of mature and immature infected RBCs in blood samples is shown. Cells are first gated according to their size (FSC-A) and granularity (SSC-A), and single cells are selected based on FSC-A and FSC-H double parameters. CD45+ cells (leukocytes) are excluded and Hoechst levels among CD71+ and CD71- CD45- RBCs are assessed. **b**- CD71- mature and CD71+ immature RBCs are gated (middle dot-plots) and the levels of Hoechst+ cells are analyzed for both CD71- normocytes (left dot-plots) and CD71+ reticulocytes (right dot-plots). An example is shown for each type of parasitized isolates and for a control donor.

reticulocytes

normocytes

*P. malariae*

*P. falciparum*

FSC-A

FSC-A

*P. vivax*

CD71

Hoechst

Hoechst

FSC-A

3.10%

0.086%

4.85%

2.36%

4.26%

0.061%

Control

FSC-A

0%

0%

0.04%

0.13%

0.17%

0.25%

**Fig.S2**: **Gating strategy of the image stream analyses of blood cells from *P. malariae* infected isolates**. Cells are gated according to their focus in bright field parameter (60 X magnification) and single cells are selected. Single cells are then gated as CD235a+ CD45- (RBCs), and next as CD71- mature and CD71+ immature RBCs. Hoechst+ and Hoechst- cells are then analyzed among mature CD71- and immature CD71+ RBCs. Percentage of each gated subsets are shown in the attached table.

**Figure S2**

**Fig. S3: Reticulocyte preference as a function of CD71 half-life**. Inferred ratio of intrinsic reticulocyte preference (*k_v_*/*k_m_*) based on the observed *Pv*:*Pm* invasion preference ratio after correcting for the one-day difference in intra-erythrocytic cycle length. Faster CD71 shedding (i.e. shorter half-lives) corresponds to an increased reticulocyte preference of *P. malariae*. The dashed blue line indicates the threshold where both species have the same invasion preference.

**Figure S3**

**Figure S4**

**Fig.S4: Gating strategy of Image stream analyses of bone marrow cells from *P. malariae* infected HIS-Hery mice**. Bone marrow cells are gated according to their focus in bright field parameter (60 X magnification) and single cells are selected. Single cells are then gated as CD235a+ CD71high and CD71low and CD71- CD235a human RBCs. The percentage of DAPI+ (DNA+) cells is analyzed in both CD235a+ CD71high and CD71low subsets.

**Figure S5**

CD235a

CD71

CD235a

CD71

Sorted cells

**a**

NS

**b**

\# Pm18S RNA copy number / 105 CD235a cells

|  | A5-2 | A5-3 | A7-6 | A7-7 | A7-9 | B7-3 | C7-4 | C7-8 |
| --- | --- | --- | --- | --- | --- | --- | --- | --- |
| CD71+ | 32.1458 | 55.4945 | 2.81033 | 0.677671 | 0.418102 | 0.176526 | 1.15283 | 2.65523 |
| CD71- | 64.0688 | 791.925 | 5.39575 | 0.593749 | 0.511204 | 0.249958 | 0 | 1.88234 |

**Fig.S5: *P. malariae* infection of bone marrow CD71+ RBCs in HIS-Hery mice**. **a**- Bone marrow cells from 9-days infected HIS-Hery mice were sorted as CD71+ and CD71- CD235a+ human RBCs cells. **b**- Copy numbers of Pm18S rRNA were quantified by qRT‑PCR in both subsets and normalized to 10⁵ CD235a+ cells (n = 9 mice). P value was found non-significant (NS) using a two-tailed paired Student’s t-test.
